# Native Collagen Preservation Versus Exogenous Collagen Addition in Xenogeneic Bone Substitutes for Dental Regeneration: Effects of Processing on Crystallinity, Remodeling, and Graft Persistence

**DOI:** 10.3390/ma19214681

**Published:** 2026-11-03

**Authors:** Claudia Valentina Pileri, Federica Monni, Manfredi Grassadonia, Chiara Cinquini, Nicola Alberto Valente

**Affiliations:** 1 Division of Periodontology, School of Dentistry, Department of Surgical Sciences, Faculty of Medicine and Surgery, University of Cagliari, Cagliari 09124, Italy; pilericv@gmail.com (C.V.P.); federicamonni1007@gmail.com (F.M.); manfredi24.grassadonia@gmail.com (M.G.); 2 Department of Surgical, Medical, Molecular and Critical Area Pathology, University of Pisa, 56126 Pisa, Italy; chiara.cinquini@unipi.it

**Keywords:** xenograft, bone substitute, collagen, bone regeneration, crystallinity, deproteinization, remodeling, biomaterials

## Abstract

**Highlights:**

- Xenograft processing can alter collagen preservation and mineral crystallinity.
- Retention of donor-derived collagen and exogenous collagen addition are distinct, potentially coexisting attributes.
- Some anorganic xenografts show slow resorption and long-term particle persistence.
- “Collagenated” alone is insufficient to characterize collagen-containing xenografts.
- NCP-XG and ECS-XG describe collagen origin and may overlap; they do not rank clinical performance.
- Graft selection should balance biological replacement with scaffold persistence.

**Abstract:**

Xenogeneic bone substitutes are widely used in dental regeneration, yet materials commonly described as collagen-containing or collagenated may differ fundamentally in their composition and biological behavior. This narrative review examines xenografts from the perspective of their manufacturing process, focusing on the distinction between preservation of native bone collagen and exogenous collagen addition, which may follow chemical or thermal processing and may also coexist with retained donor collagen. Thermal processing progressively removes the native organic matrix and carbonate components and, particularly at higher temperatures, increases mineral crystallinity and promotes the transformation of bone into a more ceramic-like scaffold. Such materials are highly osteoconductive but may exhibit very slow resorption and long-term particle persistence. Exogenous collagen can subsequently be incorporated into the processed mineral scaffold to improve cohesion, handling, and adaptation and potentially influence biological responses, but does not restore the original hierarchical mineral–collagen architecture. Conversely, chemical or enzymatic processing may preserve native collagen together with a less extensively transformed mineral phase. Available preclinical and clinical evidence suggests that some donor-collagen-retaining xenografts show higher new-bone and lower residual-graft fractions than specific anorganic comparators, although differences in animal source, porosity, mineral structure, and manufacturing protocols preclude attributing these effects exclusively to collagen preservation. We therefore use native-collagen-preserving xenografts (NCP-XG) and exogenous-collagen-supplemented xenografts (ECS-XG) as overlapping descriptors, with conformation, architecture and mineral properties recorded separately. This descriptive framework may improve interpretation of collagen-containing xenografts, but tissue fractions do not directly quantify graft replacement and collagen origin alone does not establish clinical superiority.

## 1. Introduction

Bone augmentation procedures have become an integral component of contemporary periodontal and implant therapy, where reconstruction or preservation of the alveolar process is frequently required to provide an adequate anatomical and biological environment for implant placement. Among the available grafting materials, xenogeneic bone substitutes are extensively used because of their availability, osteoconductive properties, structural similarity to human bone, and absence of donor-site morbidity. Most xenogeneic bone substitutes used in dentistry are derived from bovine, porcine, or equine bone and undergo extensive processing before clinical use to remove cellular components, lipids, potentially immunogenic proteins, and transmissible pathogens [1,2,3].

The applications considered here include alveolar ridge preservation after extraction, where the graft supports socket healing and limits loss of ridge contour; maxillary sinus floor augmentation, where it fills an elevated compartment to support bone formation before or with implant placement; and guided bone regeneration for horizontal or vertical ridge deficiencies and peri-implant dehiscence defects, where graft containment and space maintenance are central [3,4,5,6,7,8]. Xenografts may also be used in selected periodontal intrabony and furcation defects as part of a regenerative protocol, but evidence reviewed here is concentrated on ridge preservation, sinus augmentation and GBR [9]. A graft scaffold alone does not establish regeneration of the periodontal attachment apparatus. These are application contexts, not interchangeable indications for every product.

The biological significance of such processing becomes apparent when considering the structure of native bone. Bone is not simply a porous calcium-phosphate scaffold but a hierarchically organized composite in which the organic and inorganic phases are intimately integrated from the nanoscale upwards. Its extracellular matrix consists predominantly of type I collagen fibrils mineralized by nanoscale, carbonate-substituted apatite crystals, together with water and non-collagenous organic components [10,11,12]. The spatial organization and interaction of these constituents are fundamental to the mechanical and biological properties of bone, with collagen contributing toughness and structural organization while the mineral phase provides stiffness and compressive strength [10,13]. Importantly, bone mineral itself differs from highly crystalline synthetic hydroxyapatite: it consists of small, poorly crystalline, ion-substituted apatite crystals intimately associated with the collagen fibrillar network [11,12].

Transformation of animal bone into a clinically usable xenograft therefore involves an inherent balance between biological safety and preservation of the characteristics of the original tissue. Processing protocols vary considerably among commercially available xenografts and may include combinations of physical, chemical, enzymatic, and thermal treatments [1,2]. Although these procedures are primarily intended to eliminate potentially immunogenic or infectious components, they can also profoundly alter the composition, ultrastructure, porosity, surface characteristics, mineral phase, and ultimately the biological behavior of the resulting material [1,2]. Accordingly, the term xenogeneic bone substitute encompasses biomaterials that may share a common biological origin while differing substantially in their final physicochemical characteristics.

Thermal processing is particularly relevant in this respect. Heating animal bone progressively removes its organic components and, at sufficiently high temperatures, eliminates the native collagenous matrix. At the same time, increasing temperature induces modifications in the mineral phase, including loss of carbonate, crystal growth, increased crystallinity, changes in surface area and microstructure, and, at high sintering temperatures, structural shrinkage and the development of a more ceramic-like material [14,15,16]. Experimental comparison of low-temperature chemically treated bone with the same material subsequently sintered at 1100 °C demonstrated complete loss of the collagen and carbonate phases after sintering, together with increased mineral crystallinity, reduced porosity, structural shrinkage, crack formation, and a more brittle mechanical behavior [16]. Similarly, increasing the sintering temperature of bovine-derived hydroxyapatite has been associated with increased crystallite size and decreased surface area, with corresponding changes in its in vivo osteoconductive behavior [14]. Thus, thermal processing should not be regarded merely as a purification step: depending on its intensity, it can progressively transform native bone from a mineralized biological composite into an essentially anorganic calcium-phosphate ceramic. Deproteinization may also be achieved chemically without sintering; these processes should not be used interchangeably.

This transformation is particularly relevant to graft remodeling. Highly processed anorganic xenografts can provide a stable and osteoconductive scaffold, but their mineral phase may exhibit very slow degradation. Histological observations in humans have demonstrated persistence of deproteinized bovine bone particles more than a decade after maxillary sinus augmentation with a mixture of deproteinized bovine and autogenous bone, with no significant change in particle dimensions between biopsies obtained after six months and those obtained approximately 11 years later [17]. Experimental evidence also indicates that deproteinized bovine bone mineral can remain resistant to detectable resorption even under inflammatory conditions capable of inducing substantial resorption of the surrounding native bone [18]. Such findings highlight an important distinction between osteoconduction or osseous incorporation of a graft and its actual resorption, remodeling, and eventual replacement by newly formed host bone.

An alternative manufacturing philosophy is based on lower-temperature chemical or enzymatic purification aimed at removing potentially antigenic cellular and organic components while retaining part of the native extracellular matrix, including type I collagen [3,16,19]. These materials have been described as collagen-preserving bone xenografts to distinguish them from anorganic bone xenografts obtained through processes that eliminate the native organic phase [3]. Analytical investigations have confirmed that enzymatically de-antigenated equine bone may retain type I collagen in its native conformation, whereas no detectable proteins were found in a comparator anorganic bovine xenograft subjected to high-temperature processing [19]. More recent physicochemical evidence suggests that low-temperature processing can simultaneously preserve collagen and a mineral phase closer to that of native bone, whereas subsequent sintering removes collagen and carbonate and markedly increases crystallinity [16]. Donor origin, native molecular conformation and intact mineral–collagen architecture are separate attributes; findings for a tested formulation should not be extended automatically to its entire category.

The terminology becomes more complex because the presence of collagen in a final xenogeneic biomaterial does not necessarily imply preservation of the native bone collagen. Collagen may instead be added exogenously to a previously deproteinized mineral scaffold, producing materials commonly described as collagenated xenografts. Exogenous collagen may provide clinically desirable properties such as particle cohesion, hydration, handling, adaptation to the recipient site, and stabilization of the grafted mass and may influence biological responses; however, its addition to an anorganic mineral phase is conceptually distinct from preservation of the original mineral–collagen architecture of bone [3]. Consequently, the generic terms collagen-containing or collagenated xenograft may encompass distinct, potentially coexisting biomaterial attributes: a mineral scaffold from which native collagen has been removed and to which collagen is subsequently added, and a xenogeneic bone matrix in which processing has intentionally preserved endogenous collagen.

This distinction may have consequences extending beyond the simple presence or absence of collagen. Processing may influence the degree to which the original mineral–organic architecture is retained, the crystallinity and physicochemical properties of the mineral phase, and potentially its susceptibility to dissolution, cellular resorption, and biological remodeling [1,2,14,16]. Accordingly, apparently similar collagen-containing xenografts may represent markedly different biological substrates depending on whether collagen was preserved as an integral component of the original bone matrix or reintroduced after deproteinization. Added collagen does not establish the crystallinity or resorption behavior of the underlying mineral.

The aim of this narrative review is therefore to critically examine xenogeneic bone substitutes used for dental regeneration through the perspective of their processing rather than solely according to their species of origin or commercial formulation. Particular emphasis is placed on the distinction between deproteinized xenografts supplemented with exogenous collagen, independently of the mineral-processing route and chemically or enzymatically processed xenografts in which native collagen is preserved. The review explores how these different manufacturing strategies influence mineral crystallinity and physicochemical characteristics and whether these differences translate into distinct patterns of graft resorption, biological remodeling, new bone formation, and long-term persistence. Ultimately, this processing-based framework may provide a more biologically meaningful interpretation of the term collagenated xenograft and of the clinical behavior of this heterogeneous class of bone substitutes. The descriptors are not mutually exclusive and are not proposed as a validated clinical selection algorithm.

### Literature Search and Review Approach

This narrative review was informed by a structured literature search of PubMed/MEDLINE, Scopus, and Web of Science, covering each database from inception to September 2026; the last searches were conducted in September 2026. Only English-language publications were considered. Searches combined terms related to xenograft, xenogeneic bone, bone substitute, collagen, collagenated, deproteinized, deantigenated, thermal processing, sintering, crystallinity, resorption, remodeling, and graft persistence. Reference lists of relevant articles and reviews were additionally screened to identify pertinent studies. CV and FM selected the studies. NAV reviewed uncertain cases and resolved disagreements. The complete database-specific search strings were not retained; therefore, the original search strategies cannot be reproduced in full.

Particular attention was given to studies reporting the processing method, preservation or addition of collagen, physicochemical characterization, histological or histomorphometric outcomes, and longitudinal graft behavior. Experimental studies were included when they provided mechanistic information on the effects of processing on collagen preservation, mineral structure, or biological performance. Clinical evidence was primarily considered from dental applications, including alveolar ridge preservation, guided bone regeneration, and maxillary sinus augmentation. Because terminology and manufacturing information are heterogeneous and frequently proprietary, materials were classified as native-collagen-preserving or exogenous-collagen-supplemented only when the origin of collagen could be established from the available literature. The review was designed as a narrative synthesis and no quantitative meta-analysis was performed. The synthesis distinguishes same-material processing experiments, supplementation experiments and comparisons of commercial formulations. Physicochemical studies inform material attributes, animal studies provide preclinical support, and human studies inform the specific clinical endpoints tested; previous reviews provide context and are not counted as independent primary observations. No formal risk-of-bias scoring or meta-analysis was performed. Uncertain collagen conformation, architecture or mineral attributes are reported as unknown rather than inferred from a product label.

## 2. From Native Bone to Biomaterial: How Processing Determines Xenograft Properties

### 2.1. Native Bone as a Mineral–Organic Composite

The biological and physicochemical consequences of xenograft processing can only be understood in relation to the structure of the tissue from which these materials originate. As introduced above, native bone is a hierarchically organized composite in which type I collagen fibrils and a poorly crystalline, carbonate-substituted apatite phase form an integrated mineral–organic architecture rather than two independent components. Mineral nanocrystals are intimately associated with the collagen fibrillar network, and this organization contributes to both the mechanical properties and biological behavior of bone [10,11,12]. Accordingly, processing strategies that selectively remove the organic phase or modify the mineral phase do not merely “clean” the original tissue but progressively transform the nature of the material itself.

This distinction is particularly important because the term processing encompasses several procedures with different purposes. Purification, decellularization, deantigenation, deproteinization, thermal treatment, and terminal sterilization should not be considered interchangeable terms. Purification and deantigenation primarily aim to remove potentially immunogenic cellular and organic components, whereas terminal sterilization is intended to ensure microbiological safety of the final product. Depending on the manufacturing protocol, these objectives may be achieved through chemical, enzymatic, thermal, or physical approaches, often used in combination [1,2]. Each step may nevertheless alter the original tissue, and the cumulative manufacturing process ultimately determines the physicochemical characteristics of the xenograft.

### 2.2. Thermal Processing and the Progressive Transformation into a Ceramic-like Scaffold

Thermal treatment provides an effective means of eliminating organic material from xenogeneic bone, but its effects are strongly temperature-dependent. Heating initially induces protein denaturation and progressively removes the organic phase; at higher temperatures, collagen is eliminated and substantial changes occur within the mineral component itself. These include carbonate loss, crystal growth, increased crystallinity, reduced specific surface area, changes in pore architecture, and, under intense sintering conditions, shrinkage and microstructural cracking [14,15,16].

The temperature dependence of these transformations has been demonstrated experimentally in bovine bone. Elahi et al. compared untreated bone with boiled bone and bone subsequently sintered at either 550 °C or 1100 °C. High-temperature sintering almost completely removed the organic fraction and resulted in a mineral phase with a crystallinity of approximately 95%, while microcracks were observed in thermally processed specimens [15]. Similarly, De Carvalho et al. examined chemically deproteinized bovine hydroxyapatite without subsequent sintering or after sintering at 820 °C or 1200 °C. Increasing sintering temperature progressively decreased specific surface area and increased crystallite size, demonstrating that thermal treatment modifies not only the organic content but also the structural characteristics of the remaining mineral phase [14].

Particularly informative evidence comes from the direct comparison of a low-temperature chemically processed bone graft with the same material subsequently sintered at 1100 °C. The chemically treated material retained collagen and carbonate, whereas sintering removed both components, leaving predominantly calcium phosphate with increased mineral crystallinity. Sintering was additionally associated with structural shrinkage, a reduction in porosity from approximately 81% to 72%, reduced pore and strut dimensions, and crack formation around the pores [16]. Mechanical testing further demonstrated a transition towards more brittle behavior after sintering, highlighting the structural contribution of the preserved collagenous phase [16].

Sintering intensity can alter mineral structure, but there is no universal temperature-to-clinical-performance conversion. Temperature, duration, chemical pretreatment and sterilization all matter [14,15,16]. The behavior of a commercial graft must be established from its measured properties and relevant biological evidence.

### 2.3. Chemical and Enzymatic Processing and Preservation of the Native Matrix

Chemical and enzymatic processing offers a conceptually different strategy. Rather than relying primarily on high temperatures to eliminate potentially antigenic components, these protocols aim to remove cells, lipids, blood residues, and other unwanted organic constituents while limiting alteration of the extracellular bone matrix. Under appropriately controlled conditions, this approach can preserve part of the native collagenous component and maintain a less extensively modified mineral phase [16,19].

The preservation of collagen, however, should not be assumed solely on the basis of a material being described as chemically processed or processed at low temperature. The effects depend on the specific reagents, concentrations, exposure times, subsequent processing steps, and sterilization procedures. Abdelmoneim et al., for example, characterized a low-temperature deproteinized bovine xenograft throughout chemical bleaching and subsequent gamma irradiation. Chemical treatment significantly reduced collagen content, while gamma irradiation was associated with a reduction in carbonate content, illustrating that even non-sintering manufacturing procedures may alter individual components of the native matrix [20]. Thus, low-temperature processing and collagen preservation are related but should not be regarded as synonymous concepts.

Di Stefano et al. used ATR-FTIR and SDS-PAGE to characterize enzyme-deantigenated equine bone and an anorganic bovine comparator [19]. The reported protein bands supported retention of type I collagen and the authors interpreted the pattern as predominantly native collagen; no protein was detectable in the bovine comparator by the reported methods. These assays support a material-specific interpretation and do not establish complete preservation of hierarchical architecture. Øvrebø et al. separately compared chemically treated bovine bone before and after sintering using complementary spectroscopy, thermal analysis, X-ray diffraction and microscopy [16]. Their results support processing-dependent changes in collagen and mineral properties in that experimental system, not a class-wide attribution.

### 2.4. Crystallinity and Surface Characteristics as Consequences of Processing

The distinction between thermally and non-thermally processed xenografts extends beyond collagen preservation. Manufacturing procedures produce substantial heterogeneity in crystallinity, crystal dimensions, porosity, surface area, calcium-to-phosphorus ratio, and surface morphology among xenogeneic bone substitutes [2]. These parameters are interrelated: high-temperature treatment can promote crystal growth and increased crystallinity while simultaneously reducing surface area and modifying the micro- and nanostructure available for interaction with the biological environment [14,15,16].

Mineral crystallinity and collagen retention may change together under a particular protocol, but they remain distinct analytical attributes. Mineral crystallinity should be supported by diffraction or appropriate spectroscopic analysis, carbonate by chemical or spectroscopic evidence, and porosity and surface area by their own measurements [2,14,16]. Added collagen does not establish any of these mineral characteristics.

**Figure 1:**
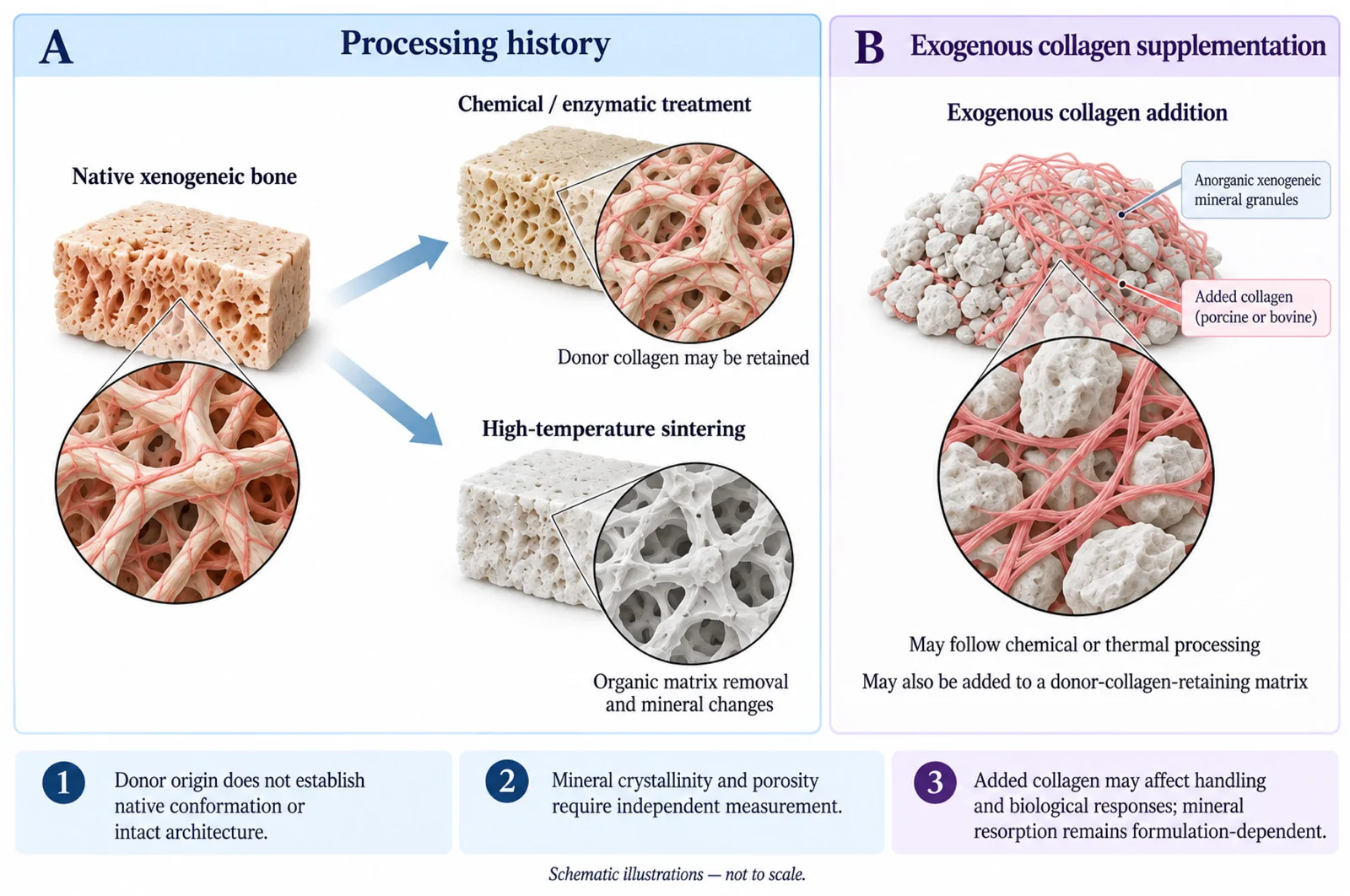
Processing history and exogenous collagen supplementation. Panel (**A**) separates chemical or enzymatic treatment from high-temperature sintering; both may change the final material, and low-temperature treatment alone does not establish native collagen conformation or intact architecture. Panel (**B**) identifies collagen supplementation as an independent formulation step, which may also coexist with retained donor collagen. The illustrations were revised with generative AI assistance from the original artwork. They are conceptual illustrations, not experimental microscopy, and are not to scale; no scale bars or remodeling–persistence ranking are used.

A defensible framework therefore records the sequence of purification, deproteinization, thermal treatment, sterilization and collagen formulation alongside measured final properties. It does not place all products on a temperature-based remodeling continuum. Figure 1 separates processing history from collagen supplementation and identifies the measurements needed to describe the final material.

## 3. Collagen in Xenogeneic Bone Substitutes: Preserved or Added?

Collagen-containing xenografts may contain donor-derived collagen, separately added collagen, or both. To make this distinction operational, we use NCP-XG for a xenogeneic bone matrix in which donor-derived collagen is retained after processing, with the strength of that attribution stated explicitly, and ECS-XG for a xenograft supplemented with separately derived collagen. NCP-XG does not, by itself, certify native molecular conformation or intact architecture. The descriptors overlap rather than forming mutually exclusive biological classes.

### 3.1. Preservation of Native Collagen

For classification, direct analytical evidence should be distinguished from a formulation description. Evidence of collagen-associated chemical groups or protein bands supports collagen detection, whereas claims about conformation require suitable structural evidence and careful interpretation. Preservation of original mineral–collagen organization requires direct architectural characterization at the scale claimed. A manufacturer description or a low processing temperature alone is insufficient to establish these properties.

The earlier CBX/ABX terminology already distinguished collagen-preserving and anorganic xenografts [3]. The additional value of NCP-XG/ECS-XG is to make collagen supplementation explicit and to permit dual labeling when donor and added collagen coexist. Each label should be accompanied by the product, evidence source and status: analytically supported, reported formulation or provisional attribution. Unknown conformation, mineral properties or processing details remain unknown. Table 1 applies this approach without extrapolating properties between brands.

### 3.2. Addition of Exogenous Collagen

A different concept underlies xenografts in which collagen is combined with an already deproteinized mineral component. Commercial formulations include deproteinized bovine or porcine bone particles combined with collagen of bovine or porcine origin, often at relatively low proportions of the final material. For example, collagenated deproteinized bovine bone mineral (DBBM) formulations containing approximately 10% porcine collagen have been developed as cohesive or moldable grafts for alveolar ridge preservation [4]. Similarly, InterOss Collagen combines bovine-derived hydroxyapatite granules with porcine-skin-derived collagen [21].

Added collagen may affect handling and biological responses. Depending on its source, concentration and crosslinking, it can change cohesion, clot retention, cellular infiltration and matrix degradation. Salamanca et al. tested collagen supplementation of a porcine mineral graft in rabbits [9]; this provides preclinical evidence for a biological effect of supplementation, not proof of native-matrix preservation. Conversely, improved initial augmented dimensions in a clinical trial of a cross-linked collagenated porcine material were followed by greater contraction during healing [5]. These observations are formulation- and endpoint-specific.

Collagen supplementation should therefore be assessed as a distinct intervention rather than treated as a surrogate for mineral crystallinity or long-term turnover. The product examples in Table 1 distinguish reported composition from directly supported structural findings. The nomenclature is descriptive and requires validation before being used predictively.

**Table 1:** Product-specific collagen attributes and the evidence supporting their description.

Product and Source	Collagen Descriptor and Integrity	Added Collagen	Processing and Mineral or Architecture Evidence	Source and Evidence Status
Bio-Oss Collagen Bovine mineral; porcine collagen	Donor collagen not retained by formulation description; ECS-XG. No class-wide conformation claim.	10% porcine collagen; non-crosslinked, as reported.	Deproteinized mineral plus added collagen. Mineral properties are not inferred from supplementation.	Clinical/formulation description [22]; comparative material report [23].
OCS-B Collagen/Straumann XenoFlex Bovine mineral; porcine collagen	ECS-XG by reported formulation. Donor-collagen integrity not independently established here.	10% porcine collagen reported in the clinical formulation.	Kim et al. explicitly identify OCS-B Collagen as Straumann XenoFlex. This does not establish equivalence of every market version or batch.	Named formulation [4]; OCS-B material comparison [21].
InterOss Collagen Bovine mineral; porcine collagen	ECS-XG by reported formulation. Intact donor architecture is not established.	10 wt.% porcine type I collagen reported.	Bovine hydroxyapatite–collagen composite; freeze-drying and dehydrothermal crosslinking reported.	Material characterization [21]; authors affiliated with manufacturer R&D.
A-Oss Collagen Bovine mineral; bovine collagen	ECS-XG by reported formulation. Native donor conformation is not established.	10% crosslinked bovine collagen reported.	Deproteinized bovine mineral plus added collagen; no crystallinity or long-term resorption assigned from this description.	Han et al. [23], final citation year 2026; first online 2025.
Osteoxenon Equine bone	NCP-XG attribution supported for the tested enzyme-deantigenated equine material.	No added collagen reported in the cited formulation.	ATR-FTIR and SDS-PAGE support collagen retention; the study authors interpret the band pattern as predominantly native. Complete hierarchical preservation is not established.	Product-specific analytical and clinical report [19]; clinical comparison [6].
OsteoBiol Gen-Os Porcine bone in the cited studies	NCP-XG by reported donor-collagen retention; conformation and full architecture are not independently established here.	No separately added collagen reported for this formulation.	Collagen-containing cortico-cancellous matrix; low-temperature processing is reported, not used alone as analytical proof.	Prior review [24] and experimental material description [25]; provisional structural attribution.
OsteoBiol mp3 Porcine bone	NCP-XG + ECS-XG by reported formulation; structural integrity remains separately qualified.	10% collagen gel reported; this is not 10% dry collagen.	Reported 90% collagen-containing granules plus 10% gel. Gel concentration and dry-collagen mass fraction are not established by that ratio. No intermediate resorption rate is inferred.	Formulation summarized in [24]; secondary description, not independent conformation evidence.

NCP-XG: donor-collagen-retaining xenograft descriptor; ECS-XG: exogenous-collagen-supplemented xenograft descriptor. Dual labeling is permitted. A reported formulation is not independent proof of native conformation or intact architecture. Percentages refer to the formulation stated in the cited source; a collagen-gel fraction is not a dry-collagen fraction. No mineral-resorption rate is assigned from the label.

## 4. Biological Dynamics: Resorption, Remodeling, and Graft Persistence

### 4.1. Resorption, Incorporation, and Replacement

Resorption is loss of graft material by cellular or physicochemical processes. Incorporation is integration of retained graft within host tissues. Replacement denotes host bone occupying space previously occupied by graft; remodeling requires evidence that removal and bone formation are coordinated. Bone-to-graft contact demonstrates osteoconductive incorporation, not necessarily replacement. Histomorphometric percentages describe the composition of a sampled region: a lower residual-graft fraction or higher new-bone fraction does not directly quantify absolute graft loss or prove that individual particles were replaced. Sampling location, tissue denominators and the amount of soft tissue also influence these proportions.

### 4.2. Persistence of Deproteinized Anorganic Xenografts

DBBM can be surrounded by newly formed bone while particles remain identifiable [26]. In a human sinus study, residual-graft fractions were 37.3% at 5 months and 20.6% at 11 months [27]. This difference is compatible with resorption during healing, but it is not a direct measurement of absolute material turnover: biopsies at different intervals sample tissue composition rather than follow the same particles continuously.

Long-term observations provide a different form of evidence. Mordenfeld et al. studied sinuses augmented with an 80:20 mixture of deproteinized bovine and autogenous bone; 11 patients underwent late biopsy at a mean of approximately 11.5 years. Residual bovine particles accounted for 17.3 ± 13.2% of sampled tissue, and particle length and area did not differ significantly from early or pristine comparators [17]. This supports long-term persistence in a mixed-graft setting, not an isolated test of DBBM alone. The inflammatory mouse experiment likewise supports resistance to detectable erosion under its specific conditions [18]. Neither observation establishes absolute non-resorbability.

### 4.3. Histological Findings with Donor-Collagen-Retaining Xenografts

Available comparative evidence suggests a different pattern for some collagen-preserving xenografts. In a randomized clinical study of sinus augmentation, enzyme-deantigenated equine bone yielded significantly more newly formed bone (46.9% vs. 25.1%) and less residual biomaterial (11.1% vs. 28.7%) after 6 months than anorganic bovine bone [6]. Similar findings were subsequently reported in extraction sockets, where enzyme-deantigenated equine bone containing preserved native type I collagen was associated with greater new bone formation and lower residual graft than the anorganic bovine comparator [19]. These product-specific comparisons do not isolate collagen retention, because species, mineral properties and manufacturing also differ.

These tissue-composition differences are consistent with different healing and persistence patterns, but they do not directly establish absolute resorption or coordinated replacement. They cannot be generalized to every NCP-XG or attributed solely to donor collagen. Such attribution would require controlled material comparisons and temporal morphological evidence.

## 5. Dental Evidence for Exogenous-Collagen-Supplemented Xenografts

### 5.1. Alveolar Ridge Preservation

Most clinical evidence for exogenous-collagen-supplemented xenografts derives from alveolar ridge preservation (ARP). Deproteinized bovine-bone mineral supplemented with approximately 10% porcine collagen (DBBM-C) has shown effective osteoconductive integration and partial preservation of post-extraction ridge dimensions [7,26,28]. Nevertheless, dimensional contraction still occurs, indicating that incorporation of collagen does not prevent physiological post-extraction remodeling [7,28].

Histomorphometric findings also demonstrate persistence of the mineral xenograft component. Gabay et al. reported 33.8% newly formed bone and 12.9% residual graft after 6 months [28]. Moreover, evaluation at different healing intervals showed increasing mineralized tissue and decreasing residual DBBM-C between 3 and 9 months, consistent with progressive maturation of the grafted site [22]. More recently, a moldable bovine mineral graft containing 10% porcine collagen also provided clinically effective ridge preservation, supporting the applicability of this material concept across different commercial formulations [4]. These tissue fractions describe sampled composition rather than direct measurement of absolute graft resorption or replacement.

### 5.2. Guided Bone Regeneration

The potential advantage of exogenous collagen in guided bone regeneration appears primarily related to graft handling and initial stability rather than to improved biological replacement. In a randomized clinical trial comparing particulate deproteinized porcine bone mineral with a cross-linked collagenated formulation, the collagenated material provided greater augmented dimensions immediately after surgery. However, this advantage disappeared after 4 months because of greater shrinkage of the collagenated graft (32.3% versus 19.9%), resulting in comparable final dimensional outcomes [5]. Dimensional contraction does not directly measure histological particle resorption.

Exogenous collagen is therefore more than a handling additive in principle, but clinical consequences depend on the formulation and outcome measured. The current comparisons do not demonstrate that supplementation recreates donor architecture, defines mineral resorption kinetics or establishes a class-wide long-term clinical advantage.

## 6. Dental Evidence for Native-Collagen-Preserving Xenografts

Clinical evidence for native-collagen-preserving xenografts is less extensive than that available for conventional anorganic xenografts, but includes applications in alveolar ridge preservation, sinus augmentation, and guided bone regeneration. In this context, histological assessment is particularly informative, since dimensional preservation alone cannot establish whether a graft has been incorporated, remodeled, or progressively replaced by newly formed bone, as also illustrated by human histological studies of alveolar ridge preservation using other classes of bone substitutes [29,30].

In extraction sockets, enzyme-deantigenated equine bone containing type I collagen in its native conformation resulted in greater newly formed bone and less residual biomaterial than a high-temperature-processed anorganic bovine xenograft [19]. A systematic review of alveolar ridge preservation similarly reported favorable histomorphometric outcomes for collagen-preserving xenografts, although substantial heterogeneity among materials and protocols limits direct comparisons [3].

Evidence from sinus augmentation supports a similar pattern. In a randomized clinical trial, enzyme-deantigenated equine bone resulted in 46.9% newly formed bone and 11.1% residual graft after 6 months, compared with 25.1% and 28.7%, respectively, for anorganic bovine bone [6]. The same observed implant survival in the two groups at 3 years did not establish statistical equivalence.

A prior narrative review describes clinical use of collagen-containing porcine grafts across several indications [24]. A small lateral-augmentation case series reported new bone around residual particles and TRAP-positive multinucleated cells [31]. TRAP positivity indicates cells with a resorptive phenotype but is not sufficient to demonstrate coordinated graft replacement. Stronger morphological support would include particle-surface erosion or resorption lacunae, spatial association with subsequent osteoid deposition and temporal evidence that host bone occupies former graft sites. Where those linked observations are unavailable, incorporation and possible resorptive activity are more defensible interpretations than proven replacement.

Overall, the available clinical literature demonstrates incorporation of particular donor-collagen-retaining formulations and differences in sampled tissue fractions in some comparisons. Limited controlled evidence and simultaneous differences in species, mineral properties and processing prevent a general causal or clinical superiority claim.

## 7. Comparative Evidence Organized by the Intervention Tested

### 7.1. Processing and Collagen Supplementation Experiments

Same-material processing experiments provide the most direct evidence that sintering changes collagen and mineral properties [14,16]. They do not establish long-term clinical efficacy. The Salamanca experiment more directly tests collagen supplementation: 20 rabbits received four calvarial defects each, including a porcine mineral graft and a composite containing the same mineral plus separately added collagen [9]. It supports a possible biological contribution of added collagen, with time- and measurement-dependent findings, and does not compare retained donor collagen with added collagen.

The Miyauchi report reanalyzed specimens from two separate rabbit experiments, rather than randomizing animals directly between the bovine and porcine products. Each source experiment used 18 rabbits, with six per healing interval; one sinus per animal was selected for this comparison. At 8 weeks, the porcine group had more new bone near the bone walls, while the other regions did not show the same between-group difference [25]. The 2-to-8-week reductions of 16.3% and 52.2% describe a histologically elevated area, not three-dimensional volume. Separate source experiments, differing materials and short follow-up restrict causal and clinical interpretation.

### 7.2. Human Commercial-Product Comparisons

The equine–bovine comparisons [6,19] address donor-collagen-retaining versus anorganic commercial formulations, with concurrent differences in species and manufacturing. The Krennmair RCT instead compares two deproteinized mineral products and does not test native versus added collagen [32]. Jung et al. compare particulate and cross-linked collagenated porcine formulations, with dimensional rather than histological endpoints [5]. These distinct questions must not be pooled into a single collagen-origin effect. Table 2 specifies each comparison, sample unit and anatomical region.

### 7.3. Evidence Hierarchy and Limits of Inference

Human randomized trials are most informative for the clinical intervention and endpoints they actually test, but modest samples, brief histological follow-up and few implant failures limit precision and long-term discrimination. Retrospective comparisons are additionally vulnerable to selection and treatment-allocation bias. Animal experiments address early healing under controlled conditions but have limited direct applicability to loaded human sites. Physicochemical studies characterize material attributes rather than clinical efficacy; previous reviews provide context and may duplicate primary evidence. None of these designs justifies a universal remodeling–persistence ranking by collagen origin.

Study-specific concerns also matter. The Krennmair analysis retained 23 of 30 randomized sinuses [32]; attrition limits precision and may affect comparison. The 2019 Di Stefano study was retrospective, and its equine material was donated by Bioteck; one author reported a scientific consultancy with that manufacturer [19]. Jain et al. were affiliated with SigmaGraft research and development [21], and the Han material-comparison study included Osstem-affiliated authors [23]. Such involvement warrants attention to design, outcome selection and independent replication, without automatically invalidating findings. These qualitative considerations are not a formal risk-of-bias assessment.

## 8. Clinical Implications: Should Processing Influence Biomaterial Selection?

The differences in composition, crystallinity, and remodeling behavior associated with xenograft processing may have clinical implications, although current evidence does not support the superiority of one processing strategy across all regenerative indications. Rather, biomaterial selection should reflect the biological and mechanical requirements of the defect. In procedures in which space maintenance and dimensional stability are major objectives, such as sinus augmentation or non-contained guided bone regeneration, persistence of the mineral scaffold may represent a desirable property. Indeed, xenogeneic grafts have demonstrated limited volumetric contraction compared with more rapidly remodeling graft materials following sinus augmentation [8].

Persistence can contribute to space maintenance, whereas resorption may permit host-tissue replacement, but faster resorption is not necessarily beneficial if it compromises the regenerative space. Histological composition, dimensional stability and clinical function are separate outcomes. Clinical benefit requires evidence on implant outcomes, complications, additional augmentation requirements or patient experience, not only tissue fractions. Current evidence does not establish the superiority of donor-collagen retention or collagen supplementation across periodontal and implant indications.

**Figure 2:**
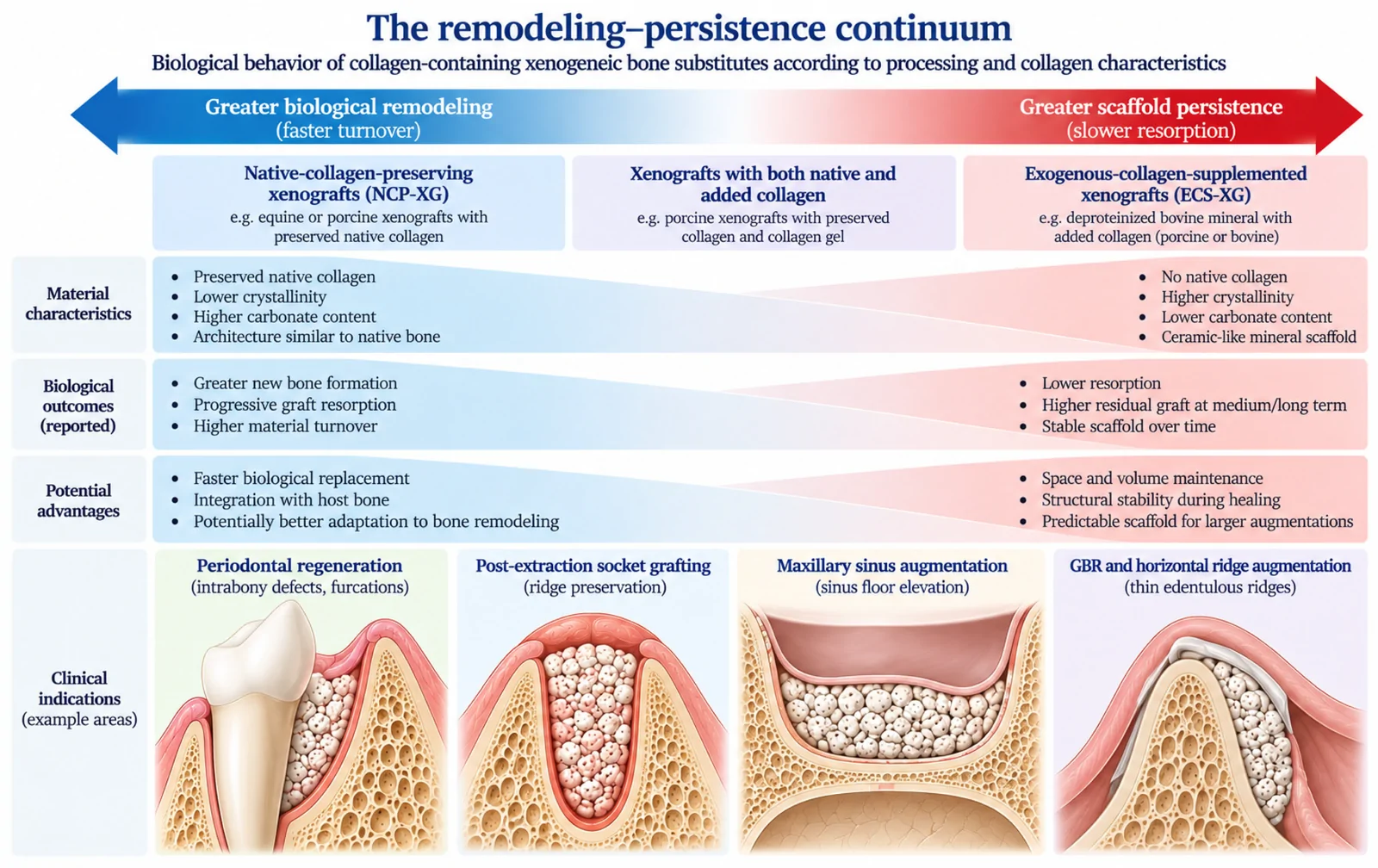
**The remodeling–persistence continuum of collagen-containing xenogeneic bone substitutes.** Xenograft processing and collagen characteristics may influence the balance between biological remodeling and scaffold persistence. Native-collagen-preserving xenografts (NCP-XG) retain a mineral–organic architecture more similar to native bone and may undergo greater biological turnover, whereas extensively deproteinized mineral scaffolds supplemented with exogenous collagen (ECS-XG) tend to exhibit slower mineral resorption and greater graft persistence. Some xenografts combine preserved native collagen with additionally incorporated collagen and therefore occupy an intermediate position. The clinical relevance of this continuum may depend on the regenerative indication and on the desired balance between biological replacement and space or volume maintenance. **The diagram represents a conceptual framework rather than a quantitative ranking of individual commercial biomaterials**.

A meta-analysis comparing bovine and porcine xenografts illustrates the limits of using animal source alone for material selection [33]. Neither species nor collagen origin substitutes for characterization of the tested formulation. Defect morphology, vascular supply, barrier and fixation strategy, required space maintenance and healing time must be considered alongside product-specific evidence. Figure 2 presents these considerations without ordering hybrid materials or clinical indications along a biological axis.

## 9. Knowledge Gaps and Future Research

Despite increasing interest in xenograft processing, several limitations currently prevent firm conclusions regarding the specific biological contribution of native collagen preservation. First, manufacturing protocols are incompletely standardized and frequently proprietary. Terms such as deproteinized, deantigenated, collagenated, and low-temperature processed are not used consistently, while processing temperatures, chemical reagents, enzymatic treatments, and terminal sterilization procedures are often insufficiently reported. Importantly, the term collagenated does not distinguish between preservation of native collagen and addition of exogenous collagen after mineral processing. The limitations of the narrative selection process and the absence of a formal risk-of-bias assessment should also be considered when interpreting the synthesis.

Direct comparisons isolating collagen origin from mineral structure, species, porosity and particle size are scarce. Small samples, short histological follow-up and region-dependent biopsies constrain interpretation. Long-term persistence data from mixed grafts cannot be treated as experiments on the mineral alone, and animal area changes should not be translated into human volume loss.

Future studies should report processing and sterilization details, donor and added collagen fractions on clearly defined wet or dry bases, conformation and architecture evidence, mineral crystallinity, carbonate, porosity and surface area. Temporal histology should seek linked evidence of erosion and subsequent bone deposition, complemented by dimensional imaging. Trials should distinguish patients, sites, biopsies and sections, report uncertainty, and include complications, additional surgery and patient-reported outcomes.

Experiments using the same mineral platform while varying donor-collagen retention, addition or crosslinking would do better to isolate causal contributions. Adequately powered clinical comparisons with longer follow-up and transparent manufacturer involvement are needed to test whether the proposed descriptors add predictive value beyond measured material properties.

## 10. Conclusions

Xenogeneic bone substitutes containing collagen should not be considered a biologically homogeneous category. Preservation of native collagen during processing and addition of exogenous collagen after deproteinization represent fundamentally different biomaterial strategies. These attributes may coexist in the same formulation and do not, by themselves, establish molecular conformation or intact architecture.

Thermal processing progressively removes the native organic matrix and carbonate components and, particularly at higher temperatures, increases mineral crystallinity and promotes a more ceramic-like scaffold. Such materials are highly osteoconductive but may undergo very slow resorption and persist within regenerated tissues for prolonged periods. Subsequent addition of exogenous collagen can improve cohesion, handling, and adaptation and may influence biological responses but does not restore the original hierarchical mineral–collagen architecture of bone.

Available experimental and clinical evidence shows higher new-bone and lower residual-graft fractions for some donor-collagen-retaining formulations than for specific anorganic comparators. These product-specific observations do not directly quantify replacement and do not isolate the contribution of collagen from species, mineral properties and other manufacturing differences.

Therefore, the clinically relevant question may not be whether one xenograft category is intrinsically superior, but which balance between scaffold persistence, dimensional stability, and biological replacement is desirable for a specific regenerative indication. More transparent reporting of manufacturing processes and carefully controlled head-to-head studies are required before processing strategy can be translated into evidence-based criteria for biomaterial selection.

## Figures and Tables

**Table 2:** Selected comparative studies grouped by the question tested and interpreted within their design limits.

Study and Sample Unit	Actual Comparison and Region	Follow-Up	Measured Findings	Interpretive Limits
Salamanca 2018 [9] Supplementation experiment 20 rabbits; four calvarial defects/animal; animal and defect counts differ.	Porcine mineral vs. same mineral + separately added type I collagen.	2, 4, 6, 8 weeks	At 8 weeks, histological NFB 21.48 ± 4.97% vs. 25.30 ± 3.51%; difference not significant. Earlier histological differences favored the composite at some intervals.	Tests supplementation, not donor retention. Multiple defects are clustered within animals. Short preclinical follow-up.
Miyauchi 2022 [25] Cross-experiment reanalysis Two source experiments, each 18 rabbits; one selected sinus/animal; n = 6/group/time.	DBBM vs. Gen-Os collagen-containing porcine bone; not a direct between-material randomized trial.	2, 4, 8 weeks	At 8 weeks, bone-wall NFB 28.6 ± 5.3% vs. 41.6 ± 6.5% (*p* = 0.004). Middle/submucosa NFB differences NS. Elevated-area reduction 2–8 weeks: 16.3% vs. 52.2%.	Bone-wall findings do not represent the entire compartment. Area is not volume. Different source experiments and materials confound comparison.
Di Stefano 2015 [6] Human sinus RCT 40 patients; 20/group; sinus biopsies at implant placement.	Enzyme-deantigenated equine bone (EDEB) vs. anorganic bovine bone (ABB).	Histology 6 months; implants 3 years	EDEB vs. ABB: NFB 46.86 ± 12.81% vs. 25.12 ± 7.25%; RG 11.05 ± 9.27% vs. 28.65 ± 9.70%; both *p* < 0.05. Observed implant survival 100% in both groups.	Patient count is not a count of histological sections. Multiple material attributes differ. Same survival percentage does not establish equivalence.
Di Stefano 2019 [19] Retrospective socket comparison 46 patients: EDEB 22, ABB 24; socket biopsies at implant placement.	EDEB vs. ABB; collagen assessed analytically in the tested materials.	Approximately 4 months	NFB 45.12 ± 10.54% vs. 33.61 ± 9.71%; RG 10.91 ± 4.27% vs. 18.47 ± 5.62%; differences reported significant (*p* < 0.05).	Retrospective selection; species and processing confounded. Manufacturer donation and consultancy disclosed. No category-wide causal inference.
Krennmair 2023 [32] Human sinus RCT 30 sinuses randomized (15/group); 23 analyzed (DPBM 10, DBBM 13).	Deproteinized porcine vs. bovine mineral; molar-region axial biopsies.	Histology 6 months; clinical 3 years post-loading	NFB 27.7 ± 5.6% vs. 22.9 ± 5.1% (*p* = 0.040). RG and graft-height reduction NS. Observed implant survival/success 100%.	Sinus sites are not patient counts. Attrition and modest samples limit precision. Does not test retained vs. added collagen or demonstrate equivalence.
Jung 2025 [5] Two-centre human GBR RCT 70 randomized; 69 analyzed (34 particulate, 35 collagenated).	Particulate DPBM vs. cross-linked collagenated DPBM; single implants with horizontal GBR in maxilla/mandible.	4 months	Hard-tissue shrinkage 19.90 ± 14.33% vs. 32.32 ± 20.79%; final dimensional differences NS. No histological NFB or RG endpoint.	Tests formulations and dimensions, not particle resorption or donor architecture. Short follow-up; one early re-entry excluded from planned analyses.

Values are mean ± SD where supplied. NFB: newly formed bone; RG: residual graft; NS: reported non-significant difference; RCT: randomized controlled trial. Patient, animal and sinus counts are distinct units and are not interchangeable with section or particle counts. Tissue fractions describe sampled regions, not absolute material resorption. Exact confidence intervals are not supplied here where they could not be verified; no precision estimates or equivalence margins have been reconstructed.

## Data Availability

No new data were created or analyzed in this study. Data sharing is not applicable to this article.

## Abbreviations

The following abbreviations are used in this manuscript:

ABX	Anorganic bone xenograft
ARP	Alveolar ridge preservation
CBX	Collagen-preserving bone xenograft
DBBM	Deproteinized bovine bone mineral
DBBM-C	Collagenated deproteinized bovine bone mineral
DPBM	Deproteinized porcine bone mineral
ECS-XG	Exogenous-collagen-supplemented xenograft
GBR	Guided bone regeneration
NCP-XG	Native-collagen-preserving xenograft
NFB	Newly formed bone
RG	Residual graft
SEM	Scanning electron microscopy
TRAP	Tartrate-resistant acid phosphatase
